# An Essay on the Origin, Development, and Eruption of the Teeth

**Published:** 1846-03

**Authors:** C. O. Cone

**Affiliations:** Fellow of the American Society of Dental Surgeons, &c.


### ARTICLE IV.

*An Essay on the Origin, Development, and Eruption of the Teeth.* Read before the Medical Society of the county of Scott, Ky. in September, 1845, by C. O. Cone, D. D. S., Fellow of the American Society of Dental Surgeons, &c. (Published by request.)

*Mr. President, and Gentlemen*
*of the Scott Co. Medical Society:*

In compliance with the requirements of the "by-laws of the society," I have prepared the following dissertation.

I shall not occupy the time of the meeting with a long introductory apology, as such apologies are usually recognized less for their sincerity than feigned modesty; and, therefore, after remarking that my hand is better trained to the exercise of my instruments than my pen, and perhaps my pen is more skilful in forming prescriptions than beautiful arranged sentences, I throw myself upon the forbearance and charity of the members of this association.

President Edwards once observed, in substance, that most of the great revolutions that have been accomplished, either in the civil, political, religious, or scientific world, were generated by zeal and perseverance. And we have, for an illustration of this fact, the names and history of Columbus. We see him rising from an examination of the Austry charts of his ancestors; then, after innumerable trials and disappointments, landing upon the shores of the western world; of Napoleon, humbly issuing from the military school of France, but soon we see his zeal and perseverance rewarded by an imperial crown, and shouts of approbation which caused the whole of Europe to fear; of John Wesley, in founding that numerous religious sect, known as the Methodists; of John Hunter, who we find in London at the age of twenty, a "green" Scotch youth, with barely sufficient education to fit him for the ordinary duties of life, becoming "the best practical anatomist of the age," and leaving a reputation which shall stand as a monument to his name, as long as his favorite science is known. But, gentlemen, notwithstanding these strong illustrations of the above observation, I hope that all the members of this society will be actuated by that zeal and perseverance, which shall make the history and doings of this association when reviewed, an additional, but a stronger illustration of President Edwards' remark.

There is no profession, so likely to bring before all its members, the organization and preservation of man, as the one which the gentlemen, who I now have the honor to address, belongs. A subject full of interest and rich in instruction, and while it directs our attention and thoughts to the Creator of all things, it is calculated to cause us to contemplate with surprise and joy, the perfection of our organization. Man consists of different organs, and these distinct organs have different purposes or offices to perform, and each, a distinct history of its organization, development and perfection.

It is to that portion of the human frame called the teeth, that I would ask your attention, and to that part of their history which relates to their origin and development ; and it is to the process of their eruption, that I would invite your special consideration.

Mr. Paget observes, that in no organs have the results of recent microscopic researches been so unexpected and so brilliant, as those connected with the teeth.* The principal originality which the author of this paper claims, is the collecting of the facts connected with these and other researches, and condensing and bringing them to that arrangement which he presents you.

It will be necessary for the proper consideration of the eruption of the teeth, to give an introductory history of the origin and development of the teeth, up to the period when eruption commences.

Most of the published views of the origin and development of the teeth, up to the days of John Hunter, were erroneous, and some supremely ridiculous. Although Mr. Hunter gives a more correct description of the formation, progress and development of the teeth, and their arrangement in the jaw, than any previous writer, still he was very incorrect in many of his opinions, and his examination into the early development of these organs very unsatisfactory.† The next writer upon this subject, who would demand our special attention, is Thomas Bell, an able and highly accomplished writer, who published his very popular work upon the teeth in 1830, in which he describes, with great care and precision, his investigations relative to the origin and development of these organs. I will not now take time to explain his opinions ; but suffice it to say, that mistake and error did attend his labors, which will be made apparent by the following epitome, (relying upon their correctness,) of the re-

* British and Foreign Medical Review, July, 1842.

† Harris' Principles and Practice of Dental Surgery, Phila. 1845.

searches of Mr. Goodsir, which are described at length, in the Edinburgh Medical and Surgical Journal, for January, 1839.

Mr. Goodsir's researches commenced at six weeks after conception; the embryo measured in length seven and a half lines, and weighed fifteen grains.

At this period, (six weeks after conception,) the jaw presented two semicircles folded around its circumference; the most external represented the true lip, the internal, the palate, and between these is a deep groove, lined by the mucous membrane of the mouth. A little later, a ridge is developed from the bottom of the groove, which looks forward, this is the rudiment of the external alveolus. At the seventh week, the germ of the first molar makes its appearance, in the aspect of a "simple, free, granular papilla" of the mucous membrane, which before was said to cover the floor of the primitive dental groove. At the eighth week, the papilla of the canine or cuspidatus tooth makes its appearanc, and, at the ninth week, the papillæ of the four incisors are seen, the two central preceding the two lateral a little; and, at the tenth week, all of the papillæ of the whole of the deciduous teeth of the upper jaw are quite distinct. The germs of the teeth in the inferior maxillary are a little more tardy in their appearance. The papilla of the first inferior molar does not appear to be any thing more than a slight bulge, at the seventh week. At about the eighth week, the primitive dental groove becomes contracted before and behind, and laminæ of mucous membrane are developed around other papillæ, which increase in growth, and enclose the papillæ in follicles with open mouths. The follicle of the first molar is completed at about the tenth week, and soon follows the completion of the follicle of the canine. At and during the eleventh and twelfth weeks, the follicles of the incisors are completed; and, at the thirteenth week, follows the posterior molar in its completion.

At about the thirteenth week, the papillæ change their forms, and assume the shapes of the teeth which they are to represent. The follicles, at the same time, have developed from their mouths small membranous processes, which act as an operculum. The opercular to each follicle corresponds to the prominences of the teeth; the incisors have two, the bicuspides three, and the molars five or more, according to their number of tubercles.

The deep portion of the groove being thus closed, as above described, by the opercula, the most elevated portion, or that nearest to the gum, is left open, and this Mr. Goodsir has named the secondary dental groove, as it serves for the development of all the permanent teeth except the molars.

At the fourteenth week, laminated inflections of mucous membrane are developed from the closing deciduous dental follicles; these lunated inflections of mucous membrane are to be the cavities of reserve for all the permanent incisors, cuspidati and bicuspides; and as the secondary dental groove is closed, they are formed into closed cavities of reserve, and recede from the surface of the gum, and lie on the inner side, and nearly in contact with the dental sacs of the deciduous teeth.

At about the fifth month, the anterior of these cavities of reserve dilates at the base, and a papilla is seen projecting into the fundus; these are the rudiments of the germs of the permanent teeth; at the same time, two small folds are produced at the superior extremity, which are to act as opercula, in converting the follicles into sacs.

At the fifth month, the posterior part of the primitive groove, behind the last deciduous tooth, is open; but, during the fifth month, it has developed within it the papilla and follicle of the first permanent molar. When this follicle becomes contracted by its operculum, there is found a large cavity of reserve, lying in contact with the dental sac on one side, and with the gum by its superficial surface. At this period, the deciduous teeth and the sacs of the ten permanent teeth increase so much in size, and there not being a proportionate lengthening of the jaw, the permanent molar is forced up and back into the maxillary *tuberosity* or *coronoid* process, which situation they occupy at the eighth and ninth month of fœtal existence.

At the age of seven or eight months, the infant jaws have so far lengthened, that the first molar has returned from its forced position in the maxillary *tuberosity*, or the *coronoid* process, to its proper position in the dental arch. The cavity of reserve, which had previously been elongated by the upward movement of the first permanent molar, now expands, and assumes the cavity and situation which the tooth last mentioned occupied when in the

*tuberosity* or *coronoid* process. A papilla is formed from its base, and the cavity becomes constricted at its superior extremity, and the dental sac of the second molar is formed, still leaving a part or portion of the cavity in connection with the superior side of the sac. As the jaw lengthens, the second permanent molar descends from its elevated position into the dental range, following the course of the first permanent molar.

The remaining part of the cavity of reserve, which was left upon the contraction of the cavity of reserve in forming the sac of the second permanent molar, has already been lengthened backward, by the ascending of the second permanent molar, again dilates, and developes a papilla and sac, as before described; and as the jaw increases in length, it follows the curve made by the first and second molars.*

It will be seen from the above, that the sacs of the permanent molar teeth do not occupy the same relation, or similar relation, to the deciduous teeth, that the ten anterior permanent teeth do.

We have now examined, briefly, the researches of Mr. Goodsir, which relate to the origin and development of the teeth of man, and which led Mr. Goodsir to form the following conclusions, to wit:

*Of the deciduous teeth:*

1st. That the milk teeth are formed on each side of the jaw at the same time, and in three distinct classes, a molar, a canine, and an incisor.

2d. That first dentition proceeds from the posterior to the anterior.

3d. That the dentition of each class advances in a reverse order, the anterior or incisors being erupted before the posterior. The first papilla that appears is the anterior molar, and the last that is completed is the posterior molar.

4th. That two of the inferior operations of dentition obey the same law that is seen to govern the classes—or this reverse law—"the follicles closing by commencing at the median line and proceeding backwards, and the dental groove disappearing in the same direction."

* Wilson's Human Anatomy.

5th. That dentition commences first in the superior maxillary, and most of the time keeps in advance.

*Of the permanent teeth:*

6th. That the papilla of the ten permanent teeth appear in a direction from the anterior to the posterior of the jaw.

7th. That all the teeth originate from the mucous membrane.

8th. That the permanent teeth have no connection with, and have a distinct origin from the temporary teeth.

Before closing this part of my paper, it may not be amiss to call your attention, for a moment, to an inaugural dissertation by Raschkow. The dissertation "relates principally to the exhibition of what he calls the adamantine organ or pulp, situated between the follicle and the germ, and destined for the production of the enamel."* Raschkow says, "the internal plane of the dental follicle is smooth, and exhibits the appearance of a serous membrane. The membrane is every where free, except where the dental germ proceeds from it.

In the internal chamber of the follicle or capsule, between its internal surface and the dental germ, another organ is also found, which, at first, almost before the first stage of the growth of the dental germ, forms nearly a globular nucleus, of which the extreme appears, in some slight degree, to bulge out, of which the internal substance presents a singular parenchyma, consisting, probably, at first, before evolution has been farther carried out, of the usual granular formative mass, similar to that of all other organs of the fœtus. Subsequently, however, by degrees, it exhibits more clear granulations of an angular form, which are connected together in various ways, by filaments of cellular tissue, and presents the appearance of a kind of *altinenchyma,* such as may be seen in plants.

This granular nucleus, which the capsular membrane surrounds, leaves a small interval between the latter, itself, and the dental germ, in which a peculiar fluid is found effused. "This fluid, when examined closely, resembles the *liquor amnii.*" The globular nucleus we have called, by anticipation, the organ of the enamel, because, from subsequent contemplation of the

*Nasmyth's Historical Introduction.

evolutions of this organ, it will be rendered clear that it is intended for the formation of the enamel substance, inasmuch as it is gradually transformed into that membrane which produces that substance.

This takes place in the following manner: the dental germ, in advancing further and further into the interior of the dental follicle, makes at first only a slight impression on the globular mass of the enamel organ; but this impression is rendered gradually deeper, as the growth of the germ proceeds. When the dental germ has penetrated further into the hollow thus made, it appears narrower towards the base, and thicker under the apex, and so is enclosed around on every side by the parenchyma of the enamel organ, which thus assumes the appearance of a hood, covering the dental germ, when advanced in its development.

This enamel hood, so to call it, presents, towards the basillary part of the dental germ, a margin, which is at first obtuse, afterwards sharp, but which is always free at every part; moreover, it still appears to be altogether as freely situated between the capsular membrane and the dental germ as previously, when it existed under the form of a granular nucleus, and it is, probably, every where surrounded by the lymph above mentioned.

When the enamel organ has assumed the form of a hood, a peculiar organ is perceived on the surface of the cavity, in which the dental germ is lodged; consisting, all of it, of short uniform fibres, placed perpendicularly to the surface of the cavity, and forming, as it were, a silky lining to the whole of the latter." According to the above quoted author, "this stratum of fibres" originates in the "transformation of the pulp of the enamel," with which it is connected at first, but soon separates itself, more and more, until no connection existed, except by some "free filaments of cellular tissue," and is converted into a true membrane, and from the office which it performs, he has named it the "membrane of the enamel."

When this membrane is closely examined, he represents its under surface to consist of hexangular, nearly uniform corpuscules, visible only through a magnifying glass, towards the centre of each of which is a rounded eminence. "Those corpuscules are nothing more than the ends of short fibres, of which the whole membrane is composed, and, which being pressed together, assume freely the hexangular form." He also describes these corpuscules as being "deposited in regular series," and corresponding with the arrangement of the enamel fibres. Each of these fibres above described as belonging to the membrane, he regards as a duct or gland, the peculiar office of which is to secrete the "enamel fibres corresponding to it." At the very commencement of the ossification of the dental pulp, each fibre, the surface of which is directed towards the subjacent bone, is brought in contact, and then commences their function, namely, secreting the earthy salts of which the enamel is principally composed. "While this is going on, an organic lymph," says Raschkow, "seems to be secreted from the parenchyma of the enamel membrane, which penetrates between the individual fibres and renders their whole substance soft."

This he thinks is by means of a "chemical organic process," afterwards combines with the earthy substances, and forms the animal basis of the enamel."*

Raschkow has shown in his paper, that the dental pulp is invested by a delicate membrane, which is said to be more vascular than the bony substance of the teeth,† and which he names the performative membrane, and believes to constitute the bond of union which exists between the enamel fibres and the bone of the tooth.‡

We have now considered three of the divisions that mark the origin and development of the teeth, to wit: the *papilla*, the follicular, and the *vascular*, sufficiently to illustrate the fourth division, the *eruptive*, to which we will now turn our attention.

There are various opinions advanced by anatomists, physiologists, and dental writers with regard to the manner or the agency by which a tooth effects its passage from its alveolar cell, and completes its eruption.

Most suppose that the tooth is raised from its alveolar cell by the elongation of the pulp and the formation of the fang, and is

* Harris' Principles and Practice of Dental Surgery.

† American Journal and Library of Dental Science, vol. 1, p. 138.

‡ Harris' Principles and Practice of Dental Surgery, Phil. 1845.

explained by a distinguished anatomist in the follow words: "As the fang grows in length, the resistance being at its end, causes the tooth to rise through the gum."* Others suppose that the alveolus is the elevating agent. That with the process of organization the tooth increases until being no longer capable of being retained in the sac, and the laws of organization recognizing the same by the close adaptation or moulting of the alveolus to the neck and fang of the increasing tooth, and effects its eruption by its own increase and its compression upon the fang of the tooth, somewhat similar to the expulsion of a moistened seed, from between the fingers by their compression.

That these explanations of the eruption of the teeth are erroneous, would appear to me to be very evident.

If we turn our attention to the early development of the teeth, and philosophically consider their advancement, we shall have not a little difficulty, in convincing ourselves, that the teeth are erupted by the resistance at the apex of the fang, resulting from the growth of the same. Did the elongation of the pulp commence before the crown had made some advancement towards the alveolus, the fang would come in contact with the floor of the alveolus, and the resistance it would then meet with, at a period when the fang is in a pulpy or yielding state, would cause the root to assume a different form from what we find presented by the fangs of a perfectly developed tooth. And we are not without illustration of the above fact. I have seen in Dr. C. A. Harris' cabinet of morbid specimens, two teeth, which from some organic difficulty, or destruction of the elevating agent, the elongation of the pulp for the formation of the fang, came in contact with the floor of the alveolus without effecting the eruption of the teeth, and resulting in the reflecting of that part, which should have constituted the fang, upon the crown of the tooth, presenting an irregular bony substance, the poorest possible apology that could be imagined for a natural tooth. I will give a description of the above referred to specimens, in Dr. Harris' words. He says, "they occupied the place of the first and second bicuspids, and their crowns are almost wholly im-

* Horner's Special Anatomy, vol. 1, p. 481.

bedded in laminated bone that should have constituted their roots, but which are entirely wanting. Judging from their appearance, one would be inclined to suppose that as they rose from their sockets, the latter filled up, and coming in contact with the former as they were forming, and while in a pulpy state, pressed against and caused them to bulge out and to be reflected upon their crowns, to the enamel of which, nearly to their grinding surfaces, they are perfectly united."*

How the tooth is to be erupted by the alveolus, I cannot easily imagine, unless the same is proved to possess contractility; powers which, I believe, none ascribe to it. But I presume that the proposition will be thought sufficiently hypothetical, to condemn it without argument.

Delabarre, a French writer of no ordinary character, has advanced a theory which is not less physiological than ingenious. He believes that the escape of the tooth from its cell and through the gum is effected precisely in the same manner, as the expulsion of the child from the uterus; and, indeed, so close does he consider the similarity, that he has given to the process of the eruption of the teeth, the "name of 'odontocie,' or accouchment of that organ."

M. Delabarre regards the sac which we have described at length, together with its relation to the tooth and the neighboring parts, in the early part of this essay, as the agent, which effects the expulsion of the tooth. "He supposes that the follicle, the vascularity of which is at that moment augmented,"† is firmly attached to the neck of the tooth, and by its contractions, the tooth is raised from its socket. This process goes on until the whole of the crown has passed through the gum, when the sac constitutes the free edge of the last named substance, which surrounds the neck of the tooth.

That the theory of Delabarre is correct, there cannot be much doubt. Mr. Goodsir, admits in his paper, that, "when the edge of the tooth has once made its way through the gum, it advances more rapidly than can be accounted for by the usual

*** Harris' Principles and Practice of Dental Surgery, p. 158,**

**† Lefoulon, Prof. Bond's translation, p. 20.**

rate of lengthening of the fang," and to believe, that this rise of the tooth which cannot be accounted for by the lengthening of the fang, is effected by the follicle, would seem to be almost a necessity, as there has not yet been found any other organ which seems destined for that purpose. It illustrates upon principles of reason and philosophy, this wonderful and beautiful operation of nature; and forms another illustration of the wisdom by which we are designed and created, and adds another demonstration of the utility, and importance, and relation of one organ to another, that

> **"Holds a link, which lost,**
> **Would break the chain, and leave behind a gap**
> **Which nature's self would rue."**

